# Scaphoid Fracture Detection and Localization Using Denoising Diffusion Models

**DOI:** 10.3390/diagnostics16010026

**Published:** 2025-12-21

**Authors:** Zhih-Cheng Huang, Tai-Hua Yang, Zhen-Li Yang, Ming-Huwi Horng

**Affiliations:** 1 Department of Computer Science and Information Engineering, National Cheng Kung University, Tainan 701, Taiwan; 2 Department of Biomedical Engineering, National Cheng Kung University, Tainan 701, Taiwan; 3 Department of Orthopedic Surgery, College of Medicine, National Cheng Kung University Hospital, National Cheng Kung University, Tainan 704, Taiwan

**Keywords:** diffusion models, anomaly detection, self-supervised, scaphoid fracture detection and localization

## Abstract

**Background/Objectives:** Scaphoid fractures are a common wrist injury, typically diagnosed and treated through X-ray imaging, a process that is often time-consuming. Among the various types of scaphoid fractures, occult and nondisplaced fractures pose significant diagnostic challenges due to their subtle appearance and variable bone density, complicating accurate identification via X-ray images. Therefore, creating a reliable assist diagnostic system based on deep learning for the scaphoid fracture detection and localization is critical. **Methods:** This study proposes a scaphoid fracture detection and localization framework based on diffusion models. In Stage I, we augment the training data set by embedding fracture anomalies. Pseudofracture regions are generated on healthy scaphoid images, producing healthy and fractured data sets, forming a self-supervised learning strategy that avoids the complex and time-consuming manual annotation of medical images. In Stage II, a diffusion-based reconstruction model learns the features of healthy scaphoid images to perform high-quality reconstruction of pseudofractured scaphoid images, generating healthy scaphoid images. In Stage III, a U-Net-like network identifies differences between the target and reconstructed images, using these differences to determine whether the target scaphoid image contains a fracture. **Results:** After model training, we evaluated its diagnostic performance on real scaphoid images by comparing the model’s results with precise fracture locations further annotated by physicians. The proposed method achieved an image area under the receiver operating characteristic curve (AUROC) of 0.993 for scaphoid fracture detection, corresponding to an accuracy of 0.983, recall of 1.00, and precision of 0.975. For fracture localization, the model achieved a pixel AUROC of 0.978 and a pixel region overlap of 0.921. **Conclusions:** This study shows promise as a reliable, powerful, and scalable solution for the scaphoid fracture detection and localization. Experimental results demonstrate the strong performance of the denoising diffusion models; these models can significantly reduce diagnostic time while precisely localizing potential fracture regions, identifying conditions overlooked by the human eye.

## 1. Introduction

Hand and wrist injuries are prevalent across all age groups, with the scaphoid bone, the largest of the eight carpal bones, located proximal to the thumb and adjacent to the forearm. Due to its unique anatomical position, the scaphoid is prone to fractures, often resulting from falls on an outstretched hand or sports-related injuries, making it one of the most common types of carpal fractures. Scaphoid fractures often present with subtle symptoms, with some patients experiencing only mild discomfort, leading to misdiagnosis as a sprain and delayed medical attention. Such delays can result in complications, including delayed healing, malunion, avascular necrosis, or osteoarthritis, significantly impairing daily functionality.

During clinical evaluation, general practitioners typically assess the injury mechanism and radial wrist tenderness. If a fracture is suspected, patients are referred for radiographic imaging to confirm the diagnosis, which may necessitate surgical intervention. The standard treatment for scaphoid fractures is screw fixation surgery, which offers a short procedure time and favorable recovery outcomes. However, this surgery requires precise localization of the scaphoid and the fracture site to ensure optimal screw placement. Clinicians typically rely on X-ray imaging for diagnosis, but variations in bone density among patients, particularly in nondisplaced fractures, can make fracture lines difficult to discern on X-ray images.

**Figure 1:**
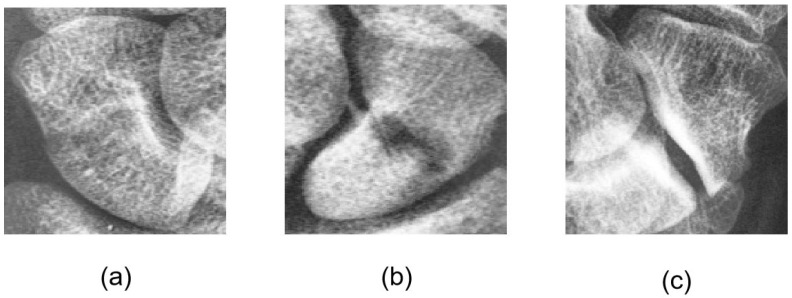
Occult fracture (**a**), nondisplaced fracture (**b**), and displaced fracture (**c**).

The difficult-to-detect fractures are termed “occult fractures.” Zander et al. [1] and Hendrix [2] indicated that occult fractures have an incidence rate of approximately 7–21%. The occult fractures, variations in bone density among patients, further complicate visual diagnosis. Consequently, clinicians may resort to computed tomography (CT) or magnetic resonance imaging (MRI) for more accurate diagnosis or surgical planning. However, these imaging modalities are costly, underscoring the importance of improving the diagnostic accuracy of X-ray imaging. Figure 1 shows scaphoid fractures that exhibit diverse patterns, including displaced, nondisplaced, and occult fractures, which pose significant challenges for precise localization of the fracture site.

The convolutional neural network had been applied to tackle the detection and classification of scaphoid in X-ray radiography. Butzow et al. [3] proposed a segmentation-based deep learning model to detect the scaphoid fractures, which obtained a sensitivity of 0.85, specificity of 0.83, and an accuracy of 0.85. Yang et al. [4] proposed a two-stage approach to first search the scaphoid area and then classify and detect the fractured area by using rotated bounding boxes. It obtained the fracture detection with an accuracy of 0.853, sensitivity of 0.735, and specificity of 0.920. Singh et al. [5] proposed a deep neural network to detect scaphoid fracture and used the Grad-CAM to represent the area of fractures. It obtained a sensitivity, specificity, and accuracy of 0.92, 0.88, and 0.90, respectively. Yang et al. [6] used anterior–posterior and lateral views to improve fracture detection and classification. The classification achieved an accuracy, recall, and precision of 0.899, 0.873, and 0.904.

The complex anatomical structure of the wrist makes accurate scaphoid localization a persistent challenge in medical image analysis. Addressing this, Hardalac et al. [7] had demonstrated the efficacy of deep learning-based object detection, particularly the Faster R-CNN architecture, in musculoskeletal radiograph analysis. These models facilitate the automated extraction of Regions of Interest (ROIs) from global X-ray views. By incorporating mechanisms such as Feature Pyramid Networks (FPNs), such systems can robustly delineate scaphoid boundaries while suppressing background interference, establishing a critical spatial prerequisite for subsequent fracture diagnosis.

To address the challenge of improving the accuracy of scaphoid fracture detection, a method based on the denoising diffusion probabilistic model (DDPM), which integrate anomaly detection concepts, and self-supervised learning is proposed. The anomaly detection methods take a common binary classification approach, which is widely applied in medical and industrial domains. Scaphoid fracture detection, as a binary classification task, falls under the umbrella of anomaly detection. Iqbal et al. [8] and Wolleb et al. [9] explored anomaly detection in medical imaging, while Zhang et al. [10] investigated anomaly detection in industrial imaging. Additionally, Via et al. [11] applied self-supervised learning frameworks to medical imaging for object detection tasks. Yang et al. [12] introduced DiffMic, which learns robust feature representations by modeling mutual information in global and local latent spaces. This model was tested on three medical classification tasks: placenta maturity grading using ultrasound images; skin lesion classification using dermoscopy images; and diabetic retinopathy grading using fundus images.

Our approach does not require precisely annotated data sets for training. The framework comprises three stages: scaphoid anomaly synthesis; scaphoid fracture reconstruction; and scaphoid fracture localization. The main contributions of this study are as follows:

- This study applies anomaly detection concepts and diffusion models [13] to scaphoid fracture detection and localization, demonstrating their suitability for scaphoid fracture classification and achieving highly accurate fracture localization. In a test set of 120 images, the best image area under the receiver operating characteristic curve (AUROC) [14] reached 0.993, indicating near-perfect identification of all fracture cases with minimal false positives. The pixel region overlap (PRO) curve achieved 0.926, reflecting effective fracture localization. The model maintains a low false-positive rate while accurately identifying most fracture regions.
- The self-supervised learning is used in scaphoid fracture detection by augmenting healthy scaphoid images to synthesize pseudofracture anomaly regions. By embedding fracture textures, we generate diverse training data to guide the network training process. This approach eliminates the need for complex and costly medical image annotation and enhances model generalizability due to diverse training data, improving the detection of various fracture types.
- The multiple time-step predictions of healthy images to guide DDPM [13] predictions enable the model to predict complete scaphoid structures at low time-steps while reconstructing healthy features at higher time-steps. The healthy image guidance improves the elimination of minor and prominent fractures, increasing the PROap curve by approximately 0.01 compared with single time-step guidance, demonstrating superior fracture localization capabilities.
- This study integrates denoising U-Net [15] and squeeze-and-excitation networks (SE-Net) [16] architectures to enhance attention to the entire scaphoid during image segmentation, effectively reducing the likelihood of misclassifying. After training, real fracture images are processed produce saliency maps that highlight fracture regions, enabling precise localization of scaphoid fractures and assisting clinicians in diagnosis.

## 2. Materials and Proposed Method

### 2.1. Experimental Materials

This study was conducted in collaboration with National Cheng Kung University Hospital (approval code: No. B-ER-110-528, approval date: 20 January 2023). The data set comprises 240 wrist X-ray images, including 160 images of participants with normal scaphoid bones and 80 images of patients with scaphoid fractures. All ages of participants ranged from 16 to 78, with the captured time from January 2023 to October 2024. All images were annotated by Dr. Tai-Hua Yang, an attending physician in the orthopedics department at National Cheng Kung University Hospital. Scaphoid images were extracted from wrist X-ray images and subjected to secondary annotation, which included identifying the presence of fractures and their approximate locations. Fracture annotations were confirmed through surgical validation, and the approximate fracture locations were manually annotated by Dr. Yang using a custom annotation tool provided by this study. These annotations may include nonfractured regions. Since this study employs segmentation to identify the precise locations of scaphoid fractures, the fracture annotations were refined and used solely for evaluating the performance of the final anomaly detection model.

The study uses normal scaphoid images from healthy patients as the training set and fractured scaphoid images from confirmed cases as the test set to evaluate the proposed method and model performance. The scaphoid images were divided into fractured and nonfractured categories. A diffusion-based anomaly reconstruction model was trained using 120 nonfractured images, and a test set of 120 images (40 nonfractured and 80 fractured) was used to evaluate the anomaly detection model.

To assess the performance of the proposed system and model, fourfold cross validation was employed. Since training relies solely on nonfractured images, the nonfractured images were randomly divided into four subsets: three subsets (120 images) for training and one subset (40 images) for testing. These 40 normal images, combined with 80 fractured images, form the test set. The cross-validation ensures that each subset participates in training and testing, enhancing the evaluation of the model’s diagnostic performance on normal scaphoid images. The experimental environment utilized an NVIDIA 4090 GPU and an Intel(R) Core(TM) i7-14700KF processor. All images in the scaphoid data set were resized to 128 × 128 pixels for the fracture detection phase. The batch size was set to 16, comprising approximately eight normal samples and eight pseudofracture samples. The model was trained for 1500 epochs using the Adam optimizer with an initial learning rate of 1 × 10^−5^. All details of the data set and training setup are summarized in Table 1.

### 2.2. Proposed Methods

The objective of this study is to develop a system that, based on the identification of the scaphoid bone in full wrist X-ray images, assists physicians in determining whether a scaphoid fracture is present and precisely localizing the fracture region. Scaphoid fractures encompass displaced, nondisplaced, and occult fractures. Displaced fractures are relatively easy to identify in X-ray images due to their prominent fracture lines and displacement. In contrast, nondisplaced and occult fractures pose significant diagnostic challenges due to variations in bone density and X-ray projection effects, often leading to inconsistent diagnoses among radiologists and orthopedic surgeons. Occult fractures, in particular, lack clear fracture lines or displacement, manifesting only as subtle cortical blurring or microcracks, further complicating detection. Physicians typically rely on observing discontinuities in the scaphoid boundary, abnormal trabecular patterns, or localized bone density changes, a process that is time-consuming and requires extensive expertise, increasing clinical challenges. In emergency settings, rapid and accurate diagnosis is critical, yet manual interpretation risks misdiagnosis due to fatigue or varying expertise. Thus, there is an urgent need for efficient, automated diagnostic tools to enhance diagnostic accuracy and efficiency.

**Figure 2:**
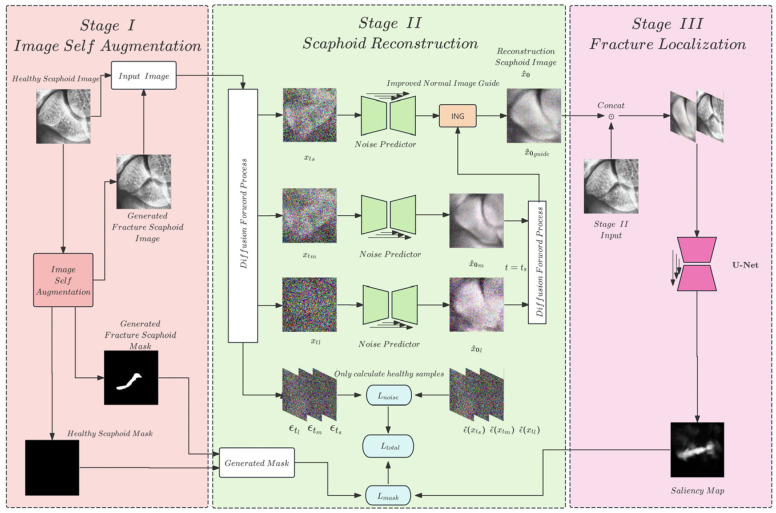
Stage I is the image self-augmentation phase. Stage II trains the scaphoid fracture reconstruction network. Stage III involves a segmentation network that identifies differences between the reconstructed images and the input images, outputting per-pixel confidence scores to produce a saliency map.

To address these challenges, this study proposes a scaphoid fracture detection and localization (ScaFDL) system. Inspired by self-supervised frameworks in medical image anomaly detection [17], we developed a framework tailored for scaphoid fracture detection. This framework integrates solutions to clinical diagnostic challenges and model training difficulties, optimizing the model structure to accommodate the complexity and diversity of scaphoid fractures. The overall model architecture is illustrated in Figure 2.

In Stage I, during the image self-augmentation process, we perform anomaly synthesis on normal scaphoid images, generating pseudoanomalies and corresponding annotations for use in training, forming a self-supervised learning framework.

In Stage II, the scaphoid fracture reconstruction network leverages the robust generative capabilities of DDPM to achieve high-quality reconstruction of fracture regions. By learning the features of normal scaphoid images, the network reconstructs pseudofractured and normal scaphoid images.

In Stage III, the fracture localization network, based on denoising U-Net and SE-Net architectures, compares subtle differences between the reconstructed images and the input images (normal or fractured) from Stage II, generating saliency maps to highlight predicted anomalous regions. These predicted regions are compared with pseudoanomaly annotations to train the model to identify pseudofracture locations, i.e., regions differing from normal images.

After training, the model can confidently localize fracture sites in real scaphoid fracture images. During testing, real fracture images are processed through Stages II and III to produce saliency maps that highlight fracture regions, enabling precise localization of scaphoid fractures and assisting physicians in diagnosis.

#### 2.2.1. Scaphoid Detection

**Figure 3:**
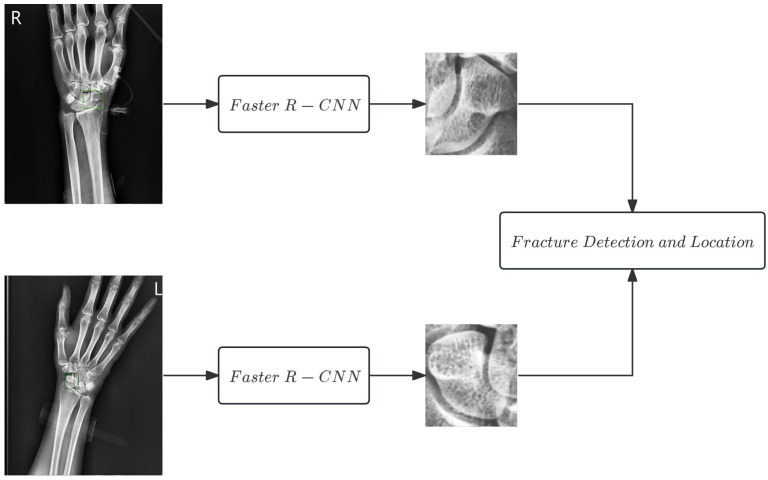
Faster R-CNN architecture used to locate the scaphoid bone in X-ray images.

The first step of this study involves isolating the scaphoid bone from the full wrist X-ray image. To achieve this, we employ faster R-CNN [18] combined with a feature pyramid network (FPN) [19]. This component, based on prior work [4], achieves robust scaphoid detection, with an intersection over union (IoU) of 0.8662 in anteroposterior views, effectively delineating the entire scaphoid. The network architecture shown in Figure 3 was adopted without significant modifications.

#### 2.2.2. Image Self-Augmentation

Annotating medical images is a significant challenge, primarily due to the time and expertise required from professional physicians as well as limitations imposed by sample scarcity, the difficulty of identifying occult fractures, and patient privacy concerns. This study aims to maximize model performance with limited training data sets. Inspired by anomaly detection models [17], we adopt image augmentation techniques to address data scarcity, reduce the need for manual scaphoid image annotation, and enhance the model’s clinical applicability. We employ a method for generating random fracture images and corresponding fracture location masks, leveraging simple image processing techniques that are cost-effective and easy to implement.

**Figure 4:**
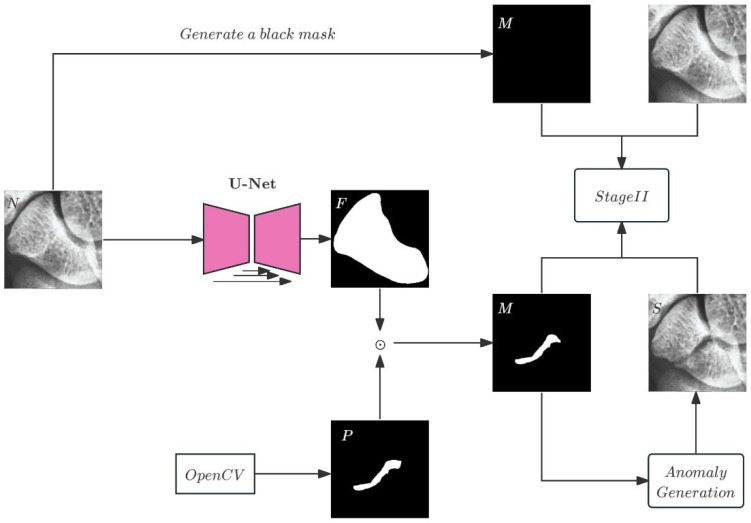
Image self-augmentation process.

To address the scarcity of annotated medical images, this study employs a fracture synthesis strategy inspired by [17], further integrating self-supervised image augmentation to generate synthetic fracture images that mimic various fracture types encountered in clinical settings. The specific steps, illustrated in Figure 4, include:

- **Scaphoid Image Preprocessing**: Prior to model input, scaphoid X-ray images undergo preprocessing, including resizing and rotation, standardized to a resolution of 128 × 128 pixels to ensure input consistency.
- **Foreground Segmentation**: Due to variations in scaphoid image size and orientation, a pretrained U-Net is used to segment the scaphoid foreground region. The U-Net processes the scaphoid X-ray image *N* to generate the corresponding foreground image *F*, as described in Equation (1). The foreground region constrains the anomaly synthesis to enhance realism, ensuring anomalies appear only within the scaphoid area. The U-Net is trained on 50 normal scaphoid images with corresponding manually annotated foreground masks and tested on 10 independent images, achieving a 98% IoU score, demonstrating excellent segmentation performance.
- **Fracture Line Simulation**: On healthy scaphoid X-ray images, linear fracture lines are randomly generated using image processing techniques. The anomaly mask *P* of Equation (1) is created by randomly generating 1 to 5 points between two endpoints, connected to form a fracture curve. The number of fracture seeds is empirically tuned to replicate the clinical heterogeneity of scaphoid injuries. Specifically, a low seed count (1–2) models the subtle, linear fissures characteristic of nondisplaced or occult fractures, whereas a higher count (3–5) generates the complex, irregular trajectories seen in displaced or comminuted patterns. This variability allows the model to learn features invariant to fracture severity, ensuring effective generalization across the full spectrum of morphological disruptions.
- **Fracture Texture Filling**: During anomaly generation, a data set of lateral view scaphoid images serves as the texture resource for filling fracture lines, as these images contain overlapping bone regions suitable for fracture textures. Additionally, manually extracted background regions from X-ray images are included in the texture data set. In Equation (2), the lateral view scaphoid image is element-wise multiplied with the anomaly mask *M* to generate a visually anomalous region *A*. This region is fused with the normal region *N* using an opacity parameter *β* to enhance visual consistency between anomalous and normal areas.
- **Image Transformations**: Transformations are applied to simulate the high variability of clinical X-ray acquisition, including exposure differences, sensor noise, and patient positioning. We implemented a rigorous augmentation pipeline incorporating both photometric and geometric perturbations:
- **Photometric Augmentation:** To simulate varying exposure and contrast conditions, we apply random Gamma correction (γ∈ [0.5,2.0] perchannel), brightness adjustment (multiplicative factor 0.8–1.2 and additive offset ±30), and Hue/Saturation shifts ±50. Additionally, stochastic Solarize (threshold “32–128”), Autocontrast, and Equalize operations are included to enhance robustness against varying bone densities.
- **Geometric Augmentation:** The input images undergo random rotation $\left(\pm 45^{\circ }\right)$. To introduce greater shape variability in the synthetic anomalies, the fracture masks undergo more aggressive transformations, including rotation $\;\left(\pm 90^{\circ }\right)$, shear (“0–40”), translation (±50% on X/Y axes), and random scaling (“50–100%”).
- During training, a stochastic selection of three augmentation techniques from this pool is applied per iteration. This strategy prevents the model from overfitting to specific synthetic artifacts and forces it to learn invariant fracture features robust to global intensity and geometric shifts.
- **Anomaly Synthesis**: The complete fracture synthesis process, detailed in Equation (2), involves the anomaly mask *M*, its inverse *M*^−1^, and element-wise multiplication ⊙. This process embeds fracture features into normal scaphoid images, producing pseudofracture images *S*.

(1) $$M=U\;\mathrm{net}\left(N\right)⊙P$$

(2) $$S=M⊙A+M^{-1}⊙N$$

The input normal image *N* is segmented by U-Net to produce the scaphoid foreground *F*. *P* represents the random fracture shape, and *M* denotes the generated fracture region, combined with the texture data set to produce the anomalous region *A*. The final pseudoanomaly sample *S* is generated. Pairs of *S* and *M* and *N* and *M* form the training set for self-supervised learning in the next stage.

Through the augmentation processes shown in Figure 4, we obtain scaphoid images with fracture features (output) and corresponding fracture location masks (mask). The random fracture generation method enables rapid synthesis of large data sets without relying on deep learning models, alleviating annotation burdens while providing initial data for subsequent diffusion-based reconstruction models, achieving multilevel data set expansion. The synthetic anomaly images, combined with healthy images, are used to train the scaphoid reconstruction network, enabling the learning of anomaly features without manual annotation. This strategy effectively scales the data set, particularly for scenarios like scaphoid fractures where annotation is challenging, significantly reducing labor costs and improving reconstruction performance.

#### 2.2.3. Scaphoid Reconstruction Network

This section details the scaphoid reconstruction module proposed in this study, corresponding to Stage II in Figure 3. The image reconstruction process leverages the powerful generative capabilities of DDPMs. However, applying DDPMs to image reconstruction, particularly for scaphoid fracture images, is challenging due to background noise, interference from other hand bones, and variations in bone density. In [20], Chan et al. explored the feasibility of using DDPMs for super-resolution skeletal image reconstruction, demonstrating that generated images closely resemble real images in visual, structural, and anatomical metrics, confirming DDPMs’ suitability for scaphoid reconstruction in medical imaging.

Additionally, Liu et al. [21] proposed a controllable image synthesis model that generates images based on text instructions or reference images. This method enables image-guided training for data sets without associated text annotations, such as our scaphoid data set. Zhang et al. applied this approach to industrial image anomaly detection, validating the feasibility of image-guided image generation [7].

According to Jiang et al. [16], the denoising and noise addition processes at different time-steps in DDPMs yield varied image generation outcomes. In our study, scaphoid fractures, including displaced, nondisplaced, and occult types, exhibit diverse sizes and shapes. Building on this insight, our method leverages the diversity of images generated at different time-steps. We generate healthy images with varying features at multiple timesteps, collectively guiding the reconstruction of healthy images through a multitime-step normal image guidance approach (Improved Normal Image Guide, ING). The detailed reconstruction process is described in the following.

The input healthy scaphoid image is denoted as $x_{0}$. We first apply noise through the forward process, as defined in Equation (3), where ${\bar{\alpha }}_{t}$ represents the noise level at time-step *t*. By adding noise of varying intensity (1 − ${\bar{\alpha }}_{t}$)*I* to $x_{0}$, we obtain noisy images $x_{t}$ for the subsequent denoising process:

(3) $$q\left(x_{t}|x_{0}\right)=N\left(x_{t};\sqrt{{\bar{\alpha }}_{t}}x_{0},\left(1-{\bar{\alpha }}_{t}\right)I\right)$$

In related work, a U-Net structure is employed for noise prediction. To meet the demands of the scaphoid reconstruction task, we enhance the original denoising U-Net structure shown by incorporating the transformer encoder from [22] into the intermediate layers, replacing the attention block with a transformer encoder. This transformer encoder consists of 12 transformer encoder layer components, each incorporating multihead self-attention mechanisms, stabilized by residual connections and layer normalization. Compared to a single-layer attention block, the transformer encoder achieves deeper feature extraction through its multilayer structure, significantly enhancing global semantic modeling. This is particularly suitable for capturing long-range dependencies in the complex anatomical structures of scaphoid reconstruction. Additionally, the transformer encoder integrates time embedding, enabling precise adaptation to the diffusion process’s timesteps and improving denoising performance.

**Figure 5:**
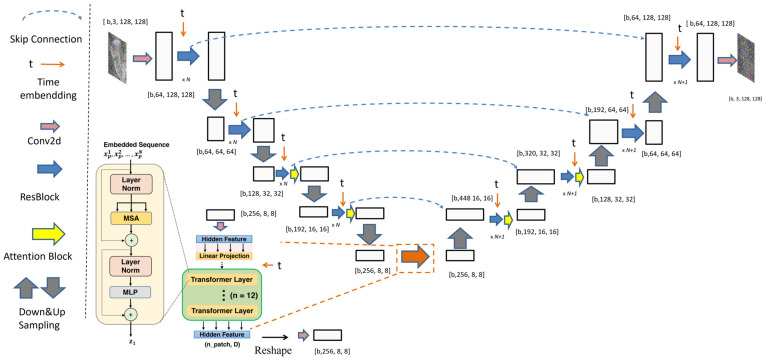
Denoising U-Net architecture with transformer integration in deep feature extraction, enhancing global semantic modeling. Time embedding is incorporated into the transformer module to precisely adapt to the diffusion process’s time-steps.

As shown in Figure 5, these improvements enhance the model’s accuracy and robustness in scaphoid reconstruction tasks, particularly in handling fine structures and maintaining global consistency. Subsequent experimental results demonstrate significant improvements in fracture classification performance.

#### 2.2.4. Multitime-Step Normal Image Guided Denoising

The improved normal image guide (ING) component utilizes normal images to guide image prediction without directly affecting the U-Net’s noise prediction process. Instead, it guides the final normal image generation by refining the predicted noise. This subsection details the two-step normal image guidance process.

We partition the time-step range *t* ∈ {0, 1, …, *T*} into three segments: short (s), medium (m), and long (l). Three random time-steps are sampled: $t_{s}$ ∈ *s*; *t_m_* ∈ *m*; and $t_{l}$ ∈ *l*. The input image $x_{0}$ is noised using Equation (4) to obtain $x_{t_{s}}$, $x_{t_{m}}$, and $x_{t_{l}}$. Based on the noise predicted by the diffusion model at $t_{m}$ and $t_{l}$, the corresponding predicted initial data $x_{0}$ is computed using Equation (4):

(4) $${\hat{x}}_{0}=\;\frac{x_{t}-\sqrt{1-{\bar{\alpha }}_{t}}\epsilon_{\theta }\left(x_{t},t\right)}{\sqrt{{\bar{\alpha }}_{t}}}$$

These ${\hat{x}}_{0}$ images are then renoised through the forward process at time-step *t_s_*, yielding $x_{t_{s}|x_{0,m}}$ and $x_{t_{s}|x_{0,l}}$. The guidance term is calculated as

(5) $$\Delta_{guided}=w_{1}\cdot (x_{t_{s}|x_{0,m}}-x_{t_{s}})+w_{2}\cdot (x_{t_{s}|x_{0,l}}-x_{t_{s}})$$

We utilize ∆*_guided_* shown in Equation (5) to guide the predicted noise at time-step *t_s_*. This guidance is controlled by two weights, *w*, where in this paper, both *w*_1_ and *w*_2_ are set to 0.5. Ultimately, the guided noise is obtained as follows:

(6) $$\epsilon_{\theta }\left(x_{t_{s}},\Delta_{guided}\right)=\epsilon_{\theta }\left(x_{t_{s}},t_{s}\right)-\sqrt{1-{\bar{\alpha }}_{t_{s}}}\cdot \Delta_{guided}$$

Finally, using Equation (6), the guided noise $\epsilon_{\theta }$($x_{t_{s}}$, ∆*_guided_*) and $x_{t_{s}}$ are used to compute the reconstructed healthy scaphoid image via a one-step denoising process (see Equation (7)):

(7) $${\hat{x}}_{0_{guide}}=\frac{{x_{t}}_{s}-\sqrt{1-{{\bar{\alpha }}_{t}}_{s}}\epsilon_{\theta }({x_{t}}_{s},\Delta_{guided})}{\sqrt{{{\bar{\alpha }}_{t}}_{s}}}$$

The two-step guidance approach refines noise across multiple time-steps, incurring higher computational costs but improving reconstruction accuracy and detail control. Multiple guidance steps capture fine structural details, making this method suitable for high-precision tasks, particularly in preserving image structure and details in high-noise data while effectively distinguishing normal and anomalous samples.

#### 2.2.5. Scaphoid Fracture Localization Network

Through the scaphoid reconstruction network, we generate high-quality healthy scaphoid images. These reconstructed images, along with corresponding fractured scaphoid images, are input into Stage III to identify differences, which is the core of Stage III.

To accurately detect these differences, this study designs a segmentation network based on the denoising U-Net architecture, comprising an encoder, decoder, and skip connections. Given the excessive noise from the background and other bone structures in scaphoid X-ray images, we incorporate SE-Net.

**Figure 6:**
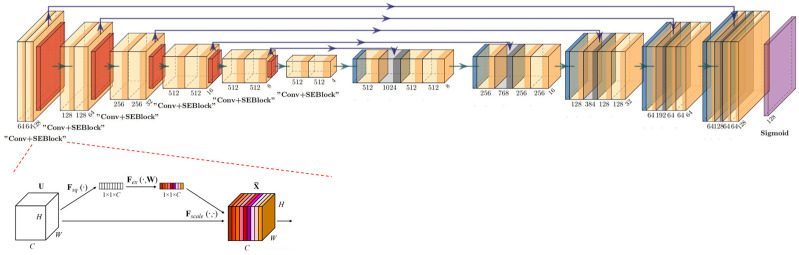
Scaphoid detection and localization network: The U-Net encoder includes an SEBlock at the end of each block. A Sigmoid function is applied at the final layer to map the output to (0, 1).

The SEBlock is added at the end of each encoder layer in the U-Net to dynamically enhance focus on the foreground scaphoid region. The network takes the concatenation of the original image *x*_0_ and the reconstructed image ${\hat{x}}_{0_{guide}}$ as input, producing a difference image as output. This output is passed through a Sigmoid layer, mapping values to (0, 1) to generate a saliency map. By deeply learning the differences and similarities between the two images while focusing on the scaphoid region, the model accurately predicts anomaly scores for each pixel in the scaphoid image (Figure 6).

#### 2.2.6. Training Loss

During the training phase, we use a total loss function to simultaneously train the scaphoid reconstruction and localization networks, with 50% normal scaphoid images and 50% pseudofracture scaphoid images as input.

For the noise prediction U-Net in the reconstruction network, only normal scaphoid data is used for training. Intuitively, this model focuses on learning the feature distribution of normal scaphoid images, minimizing the loss function $L_{\mathrm{noise}}$ to capture the overall distribution of normal images. The rationale is discussed in detail later.

One reason is that training the U-Net on pseudofracture images may lead to erroneous reconstruction of fracture features, potentially introducing bias. Livernoche et al. [23] noted that, due to cost considerations, anomaly detection models typically train on normal data sets only. If pseudofracture images are used, they must be highly realistic and anatomically accurate. Hu et al. [13] explored this, but due to suboptimal results in generating pseudofracture scaphoid images, we did not use such data sets.

Specifically, only the predicted noise from normal scaphoid image reconstruction contributes to $L_{\mathrm{noise}}$. The training minimizes the mean squared error (MSE) between the predicted noise $\epsilon_{\theta }\left(x_{t}\right)$ and the true noise $\epsilon_{t}$ across time-steps $t\in \{t_{s},t_{m},t_{l}\}$, as shown in Equation (8):

(8) $$L_{\mathrm{noise}}=\frac{1}{3}(1-y)\begin{array}{c} \sum \\ t\in \left\{t_{s},t_{m},t_{l}\right\} \end{array}{\left\|\epsilon_{t}-\epsilon_{\theta }\left(x_{t}\right)\right\|}^{2}$$

To further optimize reconstruction, we introduce the scaphoid localization network, which identifies differences $\hat{M}$ between the reconstructed and original images. This difference, along with the mask *M* generated during image augmentation, is used to compute the loss function $L_{\mathrm{mask}}$, as shown in Equation (9). This loss combines smooth L1 loss and focal loss, with *γ* as the weighting factor to balance the two losses:

(9) $$L_{\mathrm{mask}}=L_{\mathrm{smooth}}+\gamma L_{focal}$$

The smooth L1 loss and focal loss are detailed below. Equation (10) defines the smooth L1 loss, where *M* and $\hat{M}$ represent the true and predicted value matrices, respectively. *y* and *p* denote the true label (0 or 1) and predicted value [0, 1] for a single position in the matrix, and *N* is the number of samples in *M*. The smooth L1 loss considers only $\left|y-p\right|$ *<* 1:

(10) $$SmoothL1\left(y,p\right)=\left\{\begin{array}{c} 0.5\cdot {\left(y-p\right)}^{2},\;if\;\left|y-p\right|<1\\ \left|y-p\right|-0.5,\;otherwise \end{array}\right.$$

The focal loss, designed for class imbalance in classification tasks like object detection or segmentation, reduces the weight of easily classified samples to focus on challenging ones. Based on binary cross-entropy (BCE) loss [Equation (11)], it introduces two parameters: $\alpha_{t}$ and γ. $\alpha_{t}$ balances positive and negative samples, set to α= 0.75 in this study to prioritize fracture regions, which are typically smaller than normal regions. γ dynamically controls the focus, increasing from 2 to 3.5 during training to reduce the loss of easily classified samples and emphasize misclassified ones:

(11) $$BCE\left(y,p\right)=\left\{\begin{array}{c} -\log\left(p\right),\;if\;y=1\\ \log\left(1-p\right),\;if\;y=0 \end{array}\right.$$

(12) $$\alpha_{t}=\left\{\begin{array}{c} \alpha ,\;if\;y=1\\ 1-\alpha ,\;if\;y=0 \end{array}\right.$$

(13) $$p_{t}=y\cdot p+\left(1-y\right)\cdot \left(1-p\right)$$

(14) $$L_{f}\left(y,p\right)=\alpha_{t}\cdot {\left(1-p_{t}\right)}^{\gamma }\cdot \mathrm{BCE}\left(y,p\right)$$

(15) $$L_{focal}=\frac{1}{N}\sum_{i=1}^{N}L_{f}\left(y_{i},p_{i}\right)$$

The total loss function $L_{total}$ optimizes the model’s reconstruction performance for scaphoid fracture detection:

(16) $$L_{total}\;=\;L_{noise}\;+\;L_{mask}$$

## 3. Experimental Results and Discussion

### 3.1. Performance Evaluation

First, the confusion matrix shown in Table 1 is used to evaluate results. The confusion matrix is a 2 × 2 matrix, with the *x*-axis representing the ground truth and the *y*-axis indicating the model’s predictions. The outcomes are categorized as true positive (TP), false positive (FP), true negative (TN), and false negative (FN). TP indicates a correct prediction of a fracture, FP indicates a nonfractured image incorrectly predicted as fractured, TN indicates a correct prediction of a nonfractured image, and FN indicates a fractured image incorrectly predicted as nonfractured.

To evaluate the performance of scaphoid fracture detection and localization, three metrics are employed: image AUROC; pixel AUROC; and PRO. These metrics collectively provide a multidimensional evaluation framework to comprehensively assess the model’s performance across different levels.

To evaluate the performance of scaphoid fracture detection, the image AUROC is used as a primary metric. Image AUROC is a widely adopted performance measure in binary classification tasks, effectively quantifying the model’s classification capability at the image level, making it particularly suitable for scaphoid fracture detection. Image AUROC is related to the image score, calculated using Equation (17) as an image-level anomaly metric, reflecting the average intensity of anomalous regions (high-value areas) in the predicted mask. Here, $\hat{M}$ represents the predicted values for each pixel, typically in [0, 1]. The top 50 predicted values (*K* = 50) in the mask are averaged to obtain the image score.

(17) $$\mathrm{Image}\;\mathrm{Score}=\frac{1}{K}\sum_{i=1}^{K}{\mathrm{top}}_{i}\left(\mathrm{flatten}\left(\hat{M}\right)\right)$$

(18) $$\mathrm{Image}\;\mathrm{AUROC}=\mathrm{AUC}\left(Ground\;Truth,\;Image\;Score\right)$$

The image AUROC, defined in Equation (18), compares the image score across different thresholds with the ground truth labels indicating whether an image contains a fracture. The TP rate (TPR) and FP rate (FPR) are calculated using Equations (19) and (20), respectively, and the AUROC is obtained by plotting TPR against FPR and computing the area under the curve:

(19) $$TPR=\frac{TP}{TP+FN}$$

(20) $$FPR=\frac{FP}{FP+TN}$$

Additionally, pixel AUROC is used to evaluate the localization performance of scaphoid fractures, focusing on pixel-level anomaly detection. Using Equations (19) and (20), the predicted values $\hat{M}$ for each pixel across different thresholds are compared with the ground truth mask *M* to compute TPR and FPR. The area under the TPR versus FPR curve is calculated, as defined in Equation (21):

(21) $$\mathrm{Pixel}\;\mathrm{AUROC}=\mathrm{AUC}\left(flatten\left(M\right),flatten\left(\hat{M}\right)\right)$$

Since scaphoid fracture regions may occupy only a small portion of the entire scaphoid image, relying solely on pixel AUROC is insufficient for evaluating localization performance, as it primarily reflects image-level classification. To comprehensively assess fracture localization, we introduce the PRO metric, which is particularly suitable for scenarios with variable anomaly sizes and shapes. PRO focuses on the overlap between predicted and ground truth anomalous regions, making it ideal for handling irregular anomalies such as nondisplaced or occult fractures.

The core mathematical formulas for computing PRO are provided below. To convert continuous prediction scores into binary segmentation maps, a series of thresholds are applied to binarize each pixel’s predicted value, as defined in Equation (22), where [*i*,*x*,*y*] represents the pixel at position (*x*,*y*) in the *i*th image, and pred is the predicted value:

(22) $$\mathrm{Binary}\;\mathrm{Score}\;\mathrm{Maps}\left[i,x,y\right]=\left\{\begin{array}{l} 1,\;if\;pred[i,\;x,\;y]>threshold\\ 0,\;otherwise \end{array}\right.$$

The PRO metric, defined in Equation (23), is the ratio of the overlapped area between the predicted region *P* and the ground truth region *G* to the area of *G*. Note that normal images without fracture regions are excluded from PRO calculations:

(23) $$\mathrm{Region}\;\mathrm{Overlap}=\frac{P\cap G}{G}$$

The FPR, defined in Equation (24), is the ratio of the overlapped area between the predicted region *P* and the nonfractured ground truth region $\bar{G}$ to the area of $\bar{G}$:

(24) $$\mathrm{FPR}=\frac{P\cap \bar{G}}{\left|\bar{G}\right|}$$

**Figure 7:**
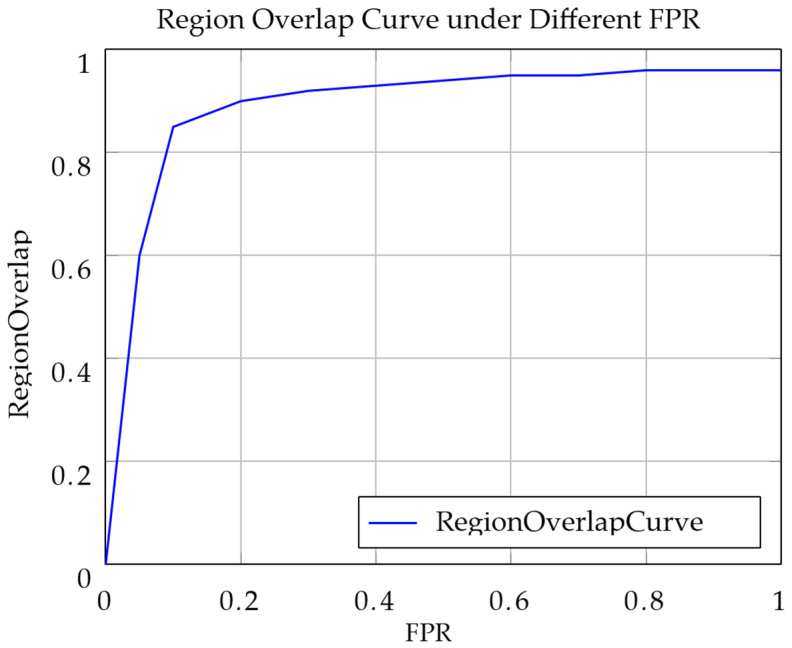
The PRO curve under different FPR values illustrates the model’s segmentation performance across thresholds. The area under the curve represents the PRO-score.

Subsequently, PRO and FPR are calculated across different thresholds. A curve is plotted with PRO against FPR, where the top-left corner represents the threshold achieving the highest recall at the lowest false positive rate, as shown in Figure 7 for a pixel PRO of 90%. However, a high FPR indicates that large normal regions are misclassified as fractured, inflating the true positive samples *TP_n_* and thus PRO values, which may not accurately reflect localization precision. Therefore, when calculating the AUC of the PRO curve, only data with FPR in the 0–30% range are considered, normalized to obtain the PRO-score, ensuring a more accurate measure of localization performance at low false positive rates.

### 3.2. Experimental Results

This section evaluates the detection and localization performance of the scaphoid fracture detection model based on the aforementioned metrics.

#### 3.2.1. Scaphoid Fracture Detection Performance

This subsection presents the final experimental results for scaphoid fracture detection.

Table 2 shows the results, demonstrating excellent performance in fracture detection (image AUROC) and localization (pixel AUROC and PRO).

The upper-left corner of the curve represents the point of optimal scaphoid detection performance, corresponding to the best threshold. Next, this paper will present the evaluation metrics at this optimal threshold along with their specific meanings.

The optimal points on the three curves are identified as follows: for image AUROC, at a threshold of 0.784, a recall of 1 is achieved with a false positive rate of 0.05; for pixel AUROC, at a threshold of 0.413, a recall of 0.952 is achieved with a false positive rate of 0.1; for PRO, at a threshold of 0.6361, a false positive rate of 0.0492 corresponds to a true overlap area of 0.8767, indicating that 87% of the fracture region is correctly identified.

#### 3.2.2. Visualization Evaluation

**Figure 8:**
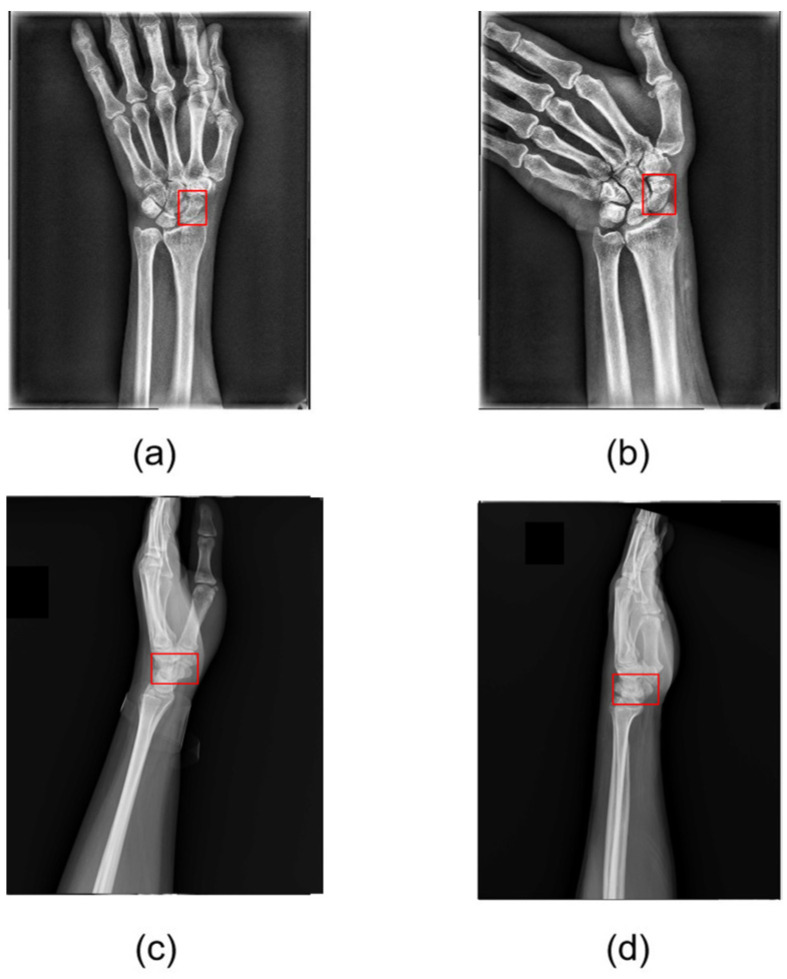
Scaphoid detection visualization: (**a**,**b**) anteroposterior and (**c**,**d**) lateral views from different patients.

This section presents the visualization results, divided into three subsections: scaphoid detection; fracture detection; and fracture localization. The image score is used to determine whether a scaphoid is fractured, with higher scores indicating a greater likelihood of fracture. The input is the scaphoid image to be analyzed, the ground truth (GT) represents the manually annotated correct fracture location, and the output mask is a saliency map indicating the detected fracture location, with pixel brightness reflecting the likelihood of a fracture. Heatmaps are generated to display fracture locations, with colors ranging from blue (low likelihood) to red (high likelihood). Figure 8 shows the results of scaphoid detection. Anteroposterior and lateral views accurately detect the scaphoid location with high confidence scores.

**Figure 9:**
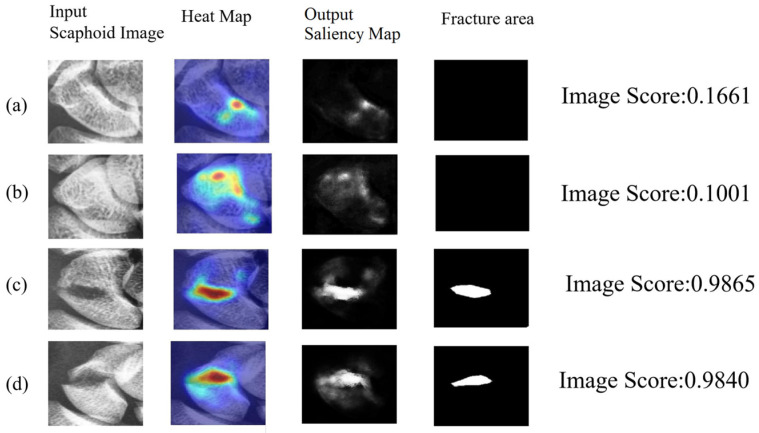
Fracture detection: The image score reflects the likelihood of a fracture. Input is the scaphoid image to be analyzed, GT is the manually annotated fracture location, output mask is the detected fracture location, and the heatmap visualizes the fracture location: (**a**,**b**) healthy scaphoids with low image scores; (**c**,**d**) fractured scaphoids with high image scores.

Figure 9 illustrates the results of scaphoid fracture detection, where the image scores enable straightforward identification of whether the scaphoid is fractured.

**Figure 10:**
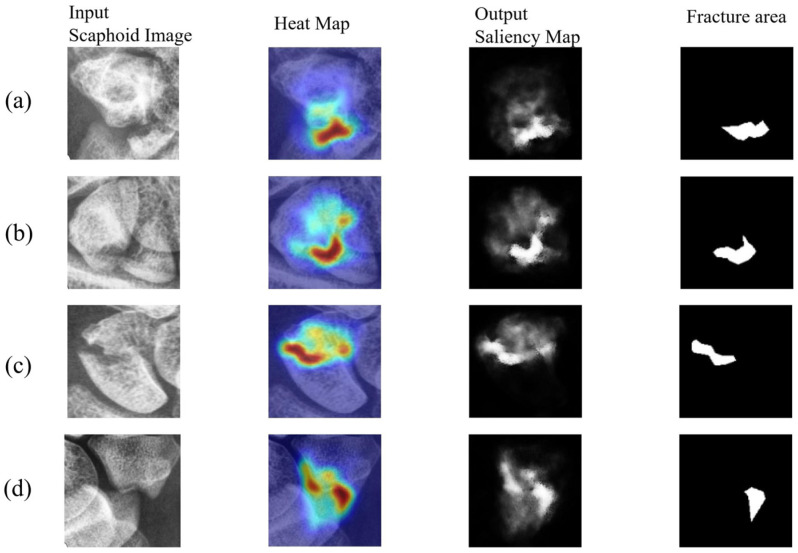
Displaced fractures: (**a**–**d**) detection results for displaced fractures from different patients. These fractures exhibit clear displacement, with image scores above 0.9, confirming fractures.

**Figure 11:**
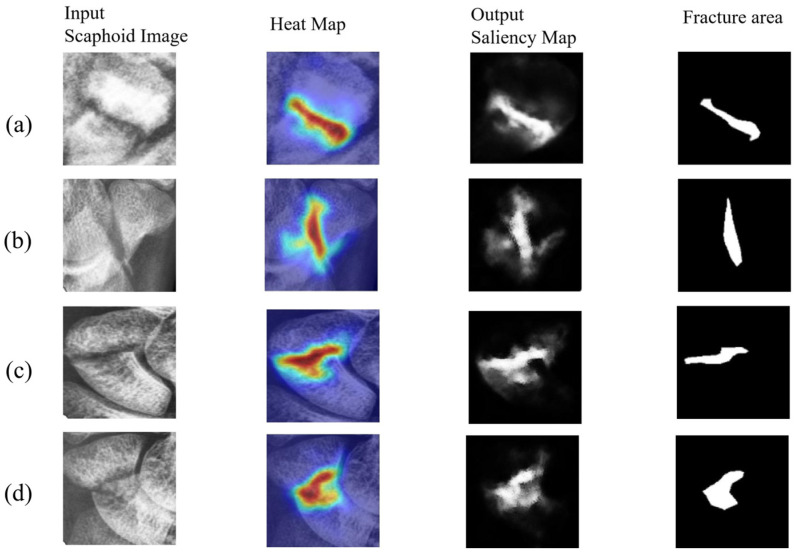
Nondisplaced fractures: (**a**–**d**) detection results for nondisplaced fractures from different patients. These fractures have clear fracture lines, with image scores above 0.95.

**Figure 12:**
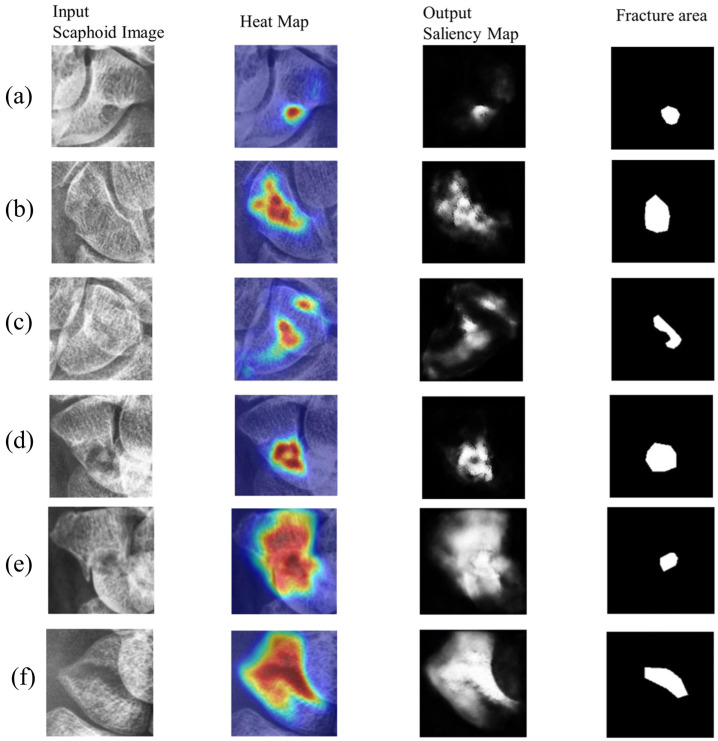
Occult fractures: (**a**–**d**) detection results for occult fractures from different patients. (**a**,**c**) have high image scores, clearly confirming fractures. (**b**,**d**) have lower scores but still identify fracture locations. Some misjudgments occur in (**a**,**b**). Finally, (**e**,**f**) depict a failure case characterized by over-segmentation, where dense physiological textures were mistaken for fracture anomalies, resulting in imprecise localization.

This subsection visualizes the results for confirmed scaphoid fracture cases, selecting three types of fractures. Figure 10 shows displaced fractures, Figure 11 shows nondisplaced fractures, and Figure 12 shows occult fractures. Figure 10 and Figure 11 demonstrate effective detection of displaced and nondisplaced fractures, accurately identifying fracture regions.

For occult fractures, as shown in Figure 12, the diversity and subtlety of fracture shapes can lead to misjudgments. However, the model generally identifies approximate fracture locations, as seen in Figure 12a,b, sufficient to alert orthopedic surgeons and expedite fracture localization. To provide a balanced performance evaluation, Figure 12e,f illustrates a representative failure case. In this instance, the model misinterprets intrinsic complex bone textures or irregular structural patterns as fracture lines. This results in a false-positive response where the predicted saliency map significantly exceeds the actual fracture boundary. This suggests that while the diffusion-based reconstruction is effective, it can occasionally struggle to differentiate between subtle fracture fissures and naturally occurring dense, irregular textures in low-contrast X-ray projections.

This subsection compares the experimental results of this study with those of other scaphoid studies, focusing on classification performance in anteroposterior views. We compare our results with those from [4] (AP view) and [6] (multiview). Our method leverages diffusion models, while prior studies used CNN-based approaches. The results, shown in Table 3, evaluate accuracy, recall, and precision.

### 3.3. Ablation Studies

This section conducts ablation studies on the proposed model, evaluating the image augmentation module, SE-Net integration, and the improved normal image guide (ING). All experiments include the image augmentation module. Subsequently, SENet and transformer structures are added, followed by an evaluation of the multitime-step ING module based on SE-Net. The test data set comprises 40 normal scaphoid images and 80 confirmed fractured images, with experiments conducted on Fold 4. A checkmark (*V*) indicates the inclusion of a module.

#### 3.3.1. Impact of SE-Net and Transformer

The model occasionally misidentifies connections between the scaphoid and other bones as fractures. To address this, SE-Net was introduced to focus on the scaphoid and reduce background interference. Table 4 shows that SE-Net improves performance by 0.01–0.02 in pixel AUROC and PRO metrics. Notably, incorporating the transformer structure in the noise prediction network improves image AUROC by 0.06, indicating significant enhancement in classification performance for distinguishing fractured and nonfractured images.

#### 3.3.2. Multitime-Step Normal Image Guidance

This subsection evaluates the ING through ablation studies, comparing three approaches: (1) traditional one-step DDPMs for scaphoid reconstruction (DDPMs, single scale); (2) DDPMs with single time-step normal image guidance (NG); and (3) DDPMs with multitime-step normal image guidance (ING). To accurately assess ING’s impact on localization performance, three test data sets are used: 100 images (40 normal + 60 fractured, primarily displaced and nondisplaced); 60 images (40 normal + 20 fractured, mostly occult); and 120 images (40 normal + 80 fractured, total).

Table 5 shows that ING significantly improves localization performance for occult fractures, with pixel PRO increasing by approximately 5%. This is attributed to multitime-step guidance providing multiscale feature information, enabling better detection of variably shaped and sized occult fractures. For other fracture types, traditional one-step DDPMs suffice for detection and localization. Computational speeds are 4.5 batches/second for DDPMs, three batches/second for NG, and two batches/second for ING.

#### 3.3.3. Visualization of Ablation Studies

**Figure 13:**
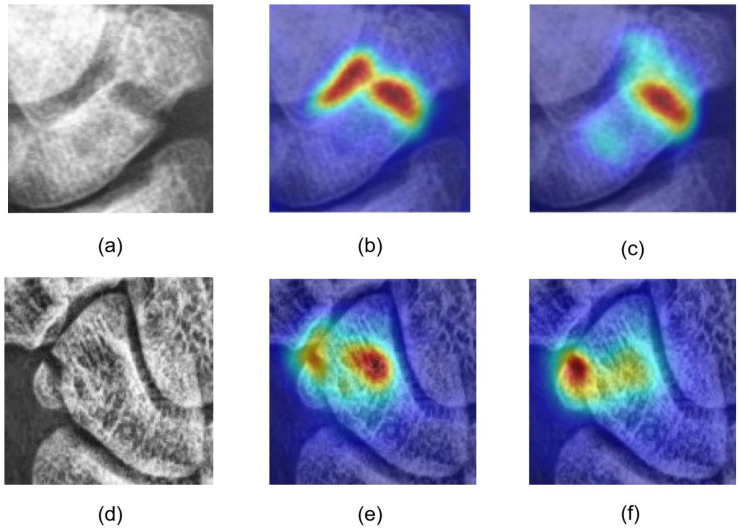
Visualization of ablation studies for different fractured scaphoids.

Figure 13 shows visualization results: (a) and (d) fractured scaphoids; (b), (c), (e), and (f) detection results at different stages; (c) incorporates foreground focusing and background suppression, reducing background misjudgments seen in (b); and (f) includes the ING module, enabling detection of subtle fractures in image corners due to multiscale feature learning, unlike (e), which fails to identify the correct fracture location.

#### 3.3.4. Comparison of Noise Levels

This subsection evaluates the impact of different time-steps on model performance. Traditional DDPMs involve 1000 noise addition and denoising steps. Smaller time-steps add less noise, resulting in reconstructed images closer to the original, potentially retaining some fractures. Larger time-steps add excessive noise, treating most structures as noise, leading to significant differences from the original image and potential misdiagnosis. This experiment aims to identify the optimal denoising time-step for the highest-quality reconstructed images.

Table 6 shows that time-steps between 300 and 500 achieve the highest model performance, indicating optimal reconstruction of normal images. The ING configuration with time-steps (300/500/700) outperforms the lowest (100/300/500) by 0.041 in PRO.

#### 3.3.5. Fine-Tuning

The model was trained on pseudofracture scaphoid data. To enhance interpretability, this subsection fine-tunes the model using real scaphoid fracture images. Table 7 shows that fine-tuning results in minimal changes to overall performance, indicating that the pseudofracture features learned by the model are comparable to real fracture features.

## 4. Discussion and Conclusions

This paper proposes an automated computer-aided scaphoid detection and localization system, i.e., a high-performance diagnostic support system that overcomes the limitations of prior studies requiring large, precisely annotated data sets. Our system requires only healthy scaphoid data sets for training, significantly reducing labor costs. By continuously learning the feature distribution of healthy scaphoid images, the system reconstructs and repairs pseudofracture scaphoid images generated through image self-augmentation. By comparing differences between pre- and post-reconstruction images, the system identifies fracture locations in pseudofracture images. Through these two stages, the model gains greater confidence in localizing fractures in real scaphoid fracture images.

During the pseudofracture image generation phase, we employed U-Net for feature extraction to constrain fracture locations to the scaphoid foreground, incorporating real fracture shape information to simulate more realistic fracture patterns. The pseudofracture features were embedded into normal scaphoid images using fracture textures, primarily to reduce medical image annotation time. Experimental results demonstrate that the simulated fracture features enable the model to learn real fracture characteristics, facilitating accurate detection of real fractures.

In the scaphoid reconstruction phase, we introduced a denoising diffusion-based scaphoid reconstruction network with multitime-step normal image guidance. DDPMs achieve robust reconstruction of scaphoid fractures, and our proposed multitime-step normal image guidance enhances reconstruction across multiple time-scales. Rather than relying on a single time-scale, the model uses images of varying quality from multiple scales to guide the reconstruction of the lowest-scale images. This approach preserves fine detail restoration at low scales while enabling reconstruction of large-scale anomalies (e.g., displaced fractures) at high scales. Through multiscale reconstruction, we obtained scaphoid images with clear contours, where both small and large fracture regions were repaired.

In the final scaphoid fracture localization phase, we utilized U-Net to identify differences between reconstructed and original fracture images, while SE-Net distinguished fracture regions from background noise. This process generated anomaly scores for each pixel, enabling the creation of heatmaps for precise fracture localization.

The proposed automated computer-aided scaphoid detection and localization system can assist physicians to rapidly diagnose the challenging scaphoid fractures. It not only confirms the presence of fractures but also predicts precise fracture locations, enabling physicians to identify potential fracture regions quickly, thus accelerating the diagnostic process. In this paper, we proposed a scaphoid fracture detection and localization method based on denoising diffusion models. However, the study meets some challenges encountered during the experimental process and related insights.

The first issue concerns the generation of pseudofracture masks. Scaphoid fractures exhibit diverse patterns, including structural changes in displaced fractures, subtle or prominent fracture lines in nondisplaced fractures, and irregular or visually imperceptible fractures in occult fractures. In our experiments, we employed simple image processing techniques to generate pseudofracture curves or lines. While this approach successfully produced pseudofracture masks, certain features remained inconsistent with real fractures.

The second issue pertains to the selection of the fracture texture data set. This study utilized lateral view fracture images and X-ray background images as the texture data set. Experimental results indicate that these textures effectively simulate real fracture patterns, enabling the model to learn partial features of real fractures from pseudofracture data. Consequently, the model achieved high diagnostic accuracy when detecting real fractures.

The third issue involves the potential for misdiagnosis. Although all images originated from the same hospital, variations in imaging parameters and environments led to inconsistent image quality, such as differences in detail, contrast, clarity, or film darkness. Variations in the distance between the arm and the sensor caused differences in scaphoid slice sizes, resulting in lower resolution after resizing. These variations could be mistaken for fractures, leading to erroneous predictions. To mitigate this, the transformations such as rotation, translation, scaling, and brightness adjustments are used to train images to simulate variations in X-ray imaging due to lighting and projection differences. Random contrast adjustments and gamma correction were also incorporated to mimic imaging variations across different equipment. These measures partially addressed image quality issues. However, when normal regions closely resembled fracture regions, the model occasionally misdiagnosed them.

The fourth issue relates to the training phase. In the scaphoid reconstruction network, we excluded pseudofracture image reconstruction from the loss function calculation. Our rationale is that including pseudofracture images could lead the model to learn fracture features, potentially treating fracture regions as normal and reconstructing them incorrectly. Thus, only the reconstruction of normal scaphoid images was included in the loss function calculation for the reconstruction network.

A primary limitation of this study is the exclusive reliance on data from a single institution, which implies potential selection bias regarding patient demographics and image acquisition protocols. Consequently, the model’s generalizability to unseen data from different scanners remains to be verified. Future work will prioritize external validation on multi-center cohorts to rigorously assess algorithmic robustness against scanner heterogeneity and varying clinical environments.

## Figures and Tables

**Table 1:** Data set and Training Setup Summary.

Feature	Count/Specification
Total X-ray Images	240
Original Composition	160 Normal Scaphoid Images
	80 Fractured Scaphoid Images
Input Resolution	128 × 128 pixels
View Distribution	Anteroposterior (AP) and Lateral Views
Reconstruction Training Set	120 Normal Images
Testing Set Size	120 Images (80 Fractured + 40 Normal)
Data Augmentation Strategy	Self-Supervised Anomaly Synthesis
Batch Size (Training)	16
Augmentation Ratio (Per Batch)	Approx. 8 Normal: 8 Pseudofracture
Validation Method	Four-fold Cross Validation

**Table 2:** Evaluation of model performance across four test data sets, with mean and standard deviation. (under 95% confidence intervals).

Test Data Sets	Image AUROC	Pixel AUROC	Pixel Region Overlap
Fold 1	0.981	0.978	0.917
Fold 2	0.994	0.978	0.924
Fold 3	0.991	0.978	0.917
Fold 4	0.993	0.980	0.926
Mean ± SD	0.9898 ± 0.0060	0.9785 ± 0.0010	0.9210 ± 0.0047

**Table 3:** Comparison of scaphoid classification performance.

Methods	Accuracy	Recall	Precision	Inference Time (FPS)	Parameters
AP view	0.853	0.789	0.894	6.24	36.7 M
Multiview	0.871	0.848	0.853	9.12	56.4 M
Ours	0.983	1.000	0.975	9.08	66.71 M

**Table 4:** Impact of SE-Net and transformer encoder on performance (under 95% confidence intervals).

SE-Net	Transformer	Image AUROC	Pixel AUROC	PRO
		0.916 ± 0.0328	0.932 ± 0.0252	0.801 ± 0.2732
*V*		0.945 ± 0.0255	0.943 ± 0.0160	0.821 ± 0.3012
*V*	*V*	0.9898 ± 0.0060	0.9785 ± 0.0010	0.9210 ± 0.0047

**Table 5:** Evaluation of normal image guidance at different time-steps in the scaphoid reconstruction phase.

Test Data	ING	Image AUROC	Pixel AUROC	Pixel Region Overlap
	DDPMs	0.978	0.981	0.893
40 + 60	NG	0.987	0.988	0.957
	ING	0.994	0.990	0.957
	DDPMs	0.910	0.904	0.787
40 + 20	NG	0.955	0.957	0.895
	ING	0.982	0.971	0.901
	DDPMs	0.978	0.948	0.835
40 + 80	NG	0.978	0.977	0.918
	ING	0.993	0.980	0.928

**Table 6:** Evaluation of ING module performance at different time-steps.

$t\in \{t_{s},t_{m},t_{l}\}$	Image AUROC	Pixel AUROC	PRO
100/300/500	0.985	0.977	0.885
200/400/600	0.981	0.978	0.917
300/400/500	0.993	0.978	0.923
300/500/700	0.993	0.980	0.926
300/600/900	0.997	0.977	0.921
400/600/800	0.994	0.978	0.924
400/700/999	0.987	0.976	0.898

**Table 7:** Fine-tuning the model with real scaphoid fracture images (under 95% confidence intervals).

Fine-Tune	Image AUROC	Pixel AUROC	Pixel Region Overlap
	0.993 ± 0.0082	0.972 ± 0.0069	0.911 ± 0.0098
V	0.993 ± 0.0071	0.980 ± 0.0052	0.926 ± 0.0076

## Data Availability

The original contributions presented in the study are included in the article, further inquiries can be directed to the corresponding author.
